# Real-time patterns of smoking and alcohol use: an observational study protocol of risky-drinking smokers

**DOI:** 10.1136/bmjopen-2014-007046

**Published:** 2015-01-06

**Authors:** Amy Cohn, Thomas Brandon, Stephen Armeli, Sarah Ehlke, Molly Bowers

**Affiliations:** 1 Schroeder Institute, Washington DC, USA; 2 Moffitt Cancer Center, University of South Florida, Tampa, Florida, USA; 3 Department of Psychology, Fairleigh Dickinson University, Teaneck, New Jersey, USA; 4 Schroeder Institute for Tobacco Research and Policy Studies, Legacy, Washington DC, USA

**Keywords:** PUBLIC HEALTH, STATISTICS & RESEARCH METHODS

## Abstract

**Introduction:**

Despite the strong relationship between smoking and health-related consequences, very few smokers quit. Heavy drinking is a significant risk factor for health consequences, and is implicated in persistent smoking and less success at quitting smoking. Self-efficacy (SE) to abstain from smoking is an important determinant of smoking outcomes and may link alcohol use to poor quit rates. Even though research has demonstrated a strong association between drinking and smoking, and the multiplicative effect of these substances on cancer-related, heavy-drinking smokers has been largely ignored in the literature. Further, research has not taken advantage of innovative methods, such as ecological momentary assessment, to capture the impact of daily factors on smoking cessation outcomes in this particular group. The proposed study identifies daily changing factors that impede or promote SE and future smoking cessation efforts in risky-drinking smokers.

**Methods and analysis:**

This is an observational study of 84 regular smokers (≥10 cigarettes per day) who drink at risky levels, report a desire to quit in the next 6 months, and show no evidence of psychiatric disturbance, severe history of alcohol withdrawal or drug dependence (excluding nicotine and caffeine). Participants report on their smoking, alcohol consumption and SE related to smoking twice a day for 28 days using interactive voice response (IVR) surveys. Multilevel regression and path models will examine within-person daily associations among drinking, smoking and SE, and how these variables predict the likelihood of future smoking behaviour at 1 and 6 months follow-up.

**Ethics and dissemination:**

This protocol was approved by an accredited Institutional Review Board. The findings will help us understand the factors that promote or impede smoking cessation among a high-risk group of smokers (heavy-drinking smokers) and will be disseminated through peer-reviewed journal articles and presentations at national conferences.

## Introduction

Cigarette smoking is one of the leading causes of cancer-related disability in the USA.^1–3^ Problematic drinking is frequently associated with persistent smoking, less success at quitting smoking and smoking relapse,^4–7^ while the concurrence of smoking and drinking represents a significant risk factor for cancer-related illness and mortality.^8–14^

**Figure 1:**
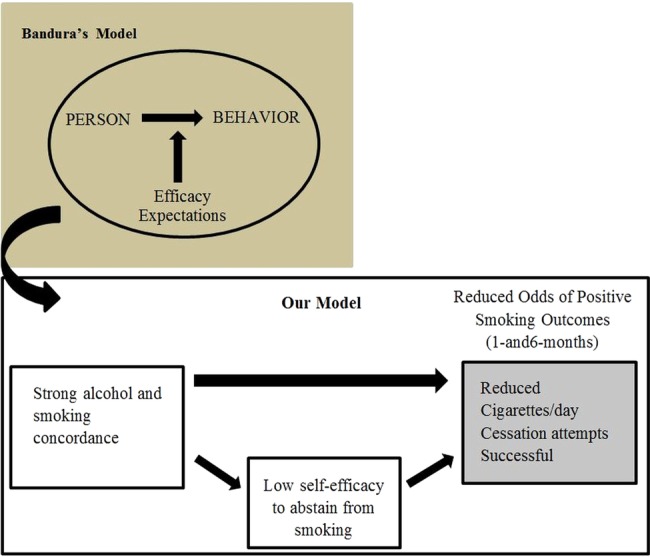
The impact of daily factors (drinking, smoking, and self-efficacy to abstain from smoking) on smoking cessation outcomes. Adapted from Bandura's^17^ model of behavior change.

Self-efficacy (SE) to quit smoking, defined as confidence in one's ability to abstain from smoking in ‘high risk’ situations, is an important determinant of smoking cessation.^15^ ^16^ According to Bandura's influential model of behaviour change^17^ (figure 1), smoking and behaviour change from smoking are influenced by SE. However, Bandura's model does not address individual differences in the co-occurrence of daily drinking and smoking, and their impact on subsequent levels of SE, even though (1) alcohol and cigarette smoking are strongly associated with one another and (2) alcohol frequently serves as a contextual trigger for smoking.^18–20^ This study expands Bandura's model by focusing explicitly on constructs at the momentary or daily level of analysis. It is hypothesised that, over and above average levels of alcohol use and smoking, individuals with stronger co-occurrence of drinking and smoking on a given day will have weaker SE at subsequent time points (ie, later that day or on the next day), and individuals with weaker SE would have a reduced likelihood of smoking behaviour change (reduced cigarettes per day, cessation attempts or successful abstinence) at follow-up.

The measurement of SE and the contextual mechanisms linking SE to smoking remain largely unspecified.^16^ ^21^ One reason is because most research has relied on retrospective measures obtained at weekly, monthly or longer intervals,^16^ which do not capture the naturally occurring events that impact SE or SE's variations across situations, contexts and days.^17^ Second, most of the previous research had not included sufficient within-person details about acute momentary changes in SE, smoking or drinking that, as purported in the literature, occur on a day-to-day basis.^22^ ^23^

One strategy for precisely measuring SE in response to everyday events is through the use of electronic diary methods, which allow for the collection of behavioural phenomena in natural settings and in, or near, real-time. These methods can establish temporal sequences among key constructs;^23–26^ they are more ecologically valid than traditional paper-and-pencil formats; and they allow for assessment of within-person variability.^27–29^ In this project, we use interactive voice response (IVR) technology, in which participants answer a set of prerecorded audio-voice questions by pushing buttons on the keypad of their telephone.

### Project aims

This study follows the daily smoking, drinking and SE patterns of a sample of 84 risky-drinking smokers. Aim 1 is to test the relationship of drinking and smoking (at the daily level) with smoking cessation outcomes at 1 and 6 months follow-up. We propose that more smoking will occur on days characterised by greater alcohol consumption (referred to as daily alcohol-smoking concordance) and that individuals with greater alcohol-smoking concordance will report worse smoking cessation outcomes.

Aim 2 is to test the mediating effect of daily SE (within days and day-to-day) on the relationship of daily alcohol-smoking concordance with short-term and long-term smoking cessation outcomes.

## Methods and analysis

### Project design

**Figure 2:**
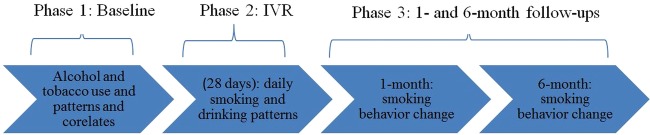
Study design and key areas of measurement at each phase of the study. IVR, interactive voice response.

The current study consists of three phases. Phase 1 is the baseline (BL) assessment, during which participants sign informed consent and complete self-report questionnaires, and gave semistructured interviews about patterns and history of cigarette and alcohol use, and related behaviours. In phase 2, participants complete twice daily IVR surveys (morning and night) for 28 days. Finally, in phase 3, participants complete a follow-up at 1 and 6 months post-BL to assess smoking and drinking since the previous assessment, motivation and desire to change (smoking and drinking), SE to quit smoking, smoking cessation attempts and the barriers to quitting (where applicable). Figure 2 displays the study design.

### Inclusion and exclusion criteria

Participants are 84 regular smokers (≥10 cigarettes per day) aged 18–65 who drink at risky levels. Risky drinking is defined according to guidelines set forth by the National Institute on Alcohol Abuse and Alcoholism: consuming >2 drinks per day for men or >1 drink per day for women; and >14 drinks per week for men or >7 drinks per week for women.^30^ The present study excludes those who report suicidal or homicidal ideation, intent or plan, or who report severe psychiatric disturbance, substance dependence with physiological dependence (excluding nicotine and caffeine), the current use of psychotropic medication for mental health, the potential for severe alcohol withdrawal (history of seizures, shakes or severe physical discomfort when absent from alcohol for at least 1 day), or who are pregnant or planning to become pregnant in the next 6 months.^i^

### Recruitment

Individuals are recruited via online advertisements, flyers, print advertisements in local newspapers and respondent driven sampling. All recruitment materials direct participants to complete an online screening or to call the study phone number to determine eligibility. Advertisements ask for research participants who smoke and drink regularly.

### Procedures

Data for this project are obtained through two sources: (1) self-report surveys and clinical interviews completed in person at BL and at 1 and 6 months post-BL, and (2) automated telephone surveys using IVR.

After an initial screening, eligible individuals are invited for a BL assessment at the study site (lasting approximately 1.5–2 h) to confirm eligibility. Before attending the BL, participants are instructed not to consume alcohol that day. At the BL, participants provide an assessment of blood alcohol concentration by expiring breath into a breathalzyer to ensure they are not intoxicated at the time of the assessment (BAC of 0.00). The informed consent document is reviewed orally at the beginning of each BL, participants are provided with a hard copy to review and they are given time to ask any questions regarding participation. A Certificate of Confidentiality (CoC) has been obtained to protect against further disclosure of the use of illegal substances or underage consumption of alcohol. A copy of the COC is available to participants on request.

Participants then complete self-report questionnaires and several interviewer-administered questionnaires about alcohol, substance use disorder and nicotine dependence symptoms as well as current alcohol, cigarette, tobacco and other drug use.

At the conclusion of the BL, participants complete a 20 min IVR training to help them better understand the survey questions. Participants are taught to record drinking data (in standard drinks) and provided the questions and response options administered by the IVR system in a paper format. They also complete a practice survey while the interviewer is in the room so that they are able to familiarise themselves with the survey and ask any questions. Participants are given a wallet-sized ‘pocket reference’ card that contains their unique subject ID (needed when completing the surveys), phone number for the study site and the date range of their IVR monitoring period. IVR monitoring begins the next morning and is scheduled to coincide with the participant's sleep-wake cycle.

### BL assessment

Measures with known psychometric properties were selected when possible. Most measures listed below are standard instruments commonly used in smoking and/or alcohol research studies.

#### Demographics and health history form

We collect information about age, race, ethnicity, income, employment and educational information, medical history (eg, diabetes, cancer, heart disease, etc), and health habits (eg, frequency of exercise and nutritional diet).

#### Prior smoking cessation experiences

Participants complete an assessment of prior smoking cessation experiences, including the number of previous quit attempts; longest (ever) quit attempt; number of quit attempts lasting at least 1 week, 1 month and 3 months; quit aids used (eg, nicotine gum, patch, etc) and reasons for relapse (eg, friends’ smoke, social pressure and life stressor).

#### Use of other tobacco products

Participants are asked about lifetime use of chewing tobacco, dip, pipe, snus, dissolvable products, hookah, little cigars/cigarillos/bidis, large cigars and e-cigarettes. Items ask about age of first use, frequency of use, period of use, and time since last use. This form was developed expressly for this study.

#### Nicotine dependence

The Fagerström Test for Nicotine Dependence (FTND^31^) is a 6-item questionnaire used to assess nicotine dependence. Respondents answer yes/no to questions about difficulty to refrain from smoking, frequency of smoking after awakening and smoking while ill. Participants also answer three categorical questions about how soon after awakening they smoke, which cigarette they would most hate to give up and how many cigarettes they smoke per day. A total score is calculated with scores ranging from 0 to 10. The FTND has demonstrated high reliability, strong validity and good internal consistency in samples of daily smokers.^32^

#### Alcohol and substance use disorder diagnoses

The Structured Clinical Interview for (Diagnostic and Statistical Manual of Mental Disorders, Fourth Edition) DSM-IV (SCID^33^) is used to assess lifetime and current diagnoses for substance and alcohol use disorders. Sections have been modified to obtain information specific to the diagnosis of nicotine dependence.

#### Motivation to change smoking

The Contemplation Ladder (CL^34^ ^35^) is a single item questionnaire designed to assess one's level of contemplation about quitting smoking on a 0 to 10 scale, where 0=*no thoughts of quitting* and 10=*taking action to quit*. The CL has shown good convergent validity with other measures of motivation to change and predicts longer term readiness to quit smoking.^35^ ^36^

The Readiness to Change Questionnaire (RTCQ^37^) is a 15-item measure that assesses readiness to change current smoking patterns based on stages of change model (precontemplation, contemplation and action). This measure is adapted from a similar measure that examines readiness to change alcohol use. Participants respond to each item using a five-point scale where −2=*strongly disagree* and 2=*strongly agree*. This measure has shown good internal and test–retest reliability in excessive drinkers.^38^

#### Motivation to change drinking

The Stages of Change and Treatment Readiness Scale (SOCRATES^39^) is a 19-item questionnaire that is used to assess readiness to change drinking and drug use habits. All responses are measured on a five-point Likert scale where 1=*strongly disagree* and 5=*strongly agree*. This measure is made up of three subscales: *Take steps*, measures of how much a person is already making changes to their alcohol or drug use; *Recognition* assesses whether a person acknowledges that they have a problem with alcohol or drugs; *Ambivalence* assesses if the person is contemplating whether or not they have an alcohol or drug problem. This measure demonstrates excellent test–retest reliability in drinkers and good convergent validity with measures of longer term drinking.^39^

#### SE to quit smoking

The Smoking Self-Efficacy Scale (SSE^40^) is a 9 item measure that assesses confidence in the ability to abstain from smoking in certain high-risk situations. Respondents use a five-point Likert scale to answer questions about how tempted they would be to smoke in certain scenarios where 1=*not at all tempted* and 5=*extremely tempted*. This measure is comprised of three subscales. The *Positive/Social* scale assesses how tempted a person may be to smoke in social settings. The *Negative/Affective* scale assesses how tempted a person may be to smoke when experiencing negative feelings. The *Habit/Addictive* scale assesses how tempted a person is to smoke in situations where many smokers use cigarettes. A total score may also be calculated to examine overall SE, with higher scores indicating lower SE. The SSE has demonstrated good reliability in the current smokers and drinkers seeking treatment.^40^

#### Cigarette, alcohol and other drug use consumption patterns

*The Time-Line Follow-Back Interview* (TLFB^41^) yields indices for quantity and frequency of alcohol, cigarette, drug and other tobacco use in the 90 days prior to the BL assessment. The TLFB has shown high test–retest reliability, and strong correlations between participant and collateral reports of drinking and smoking.^42^ ^43^

#### Alcohol use treatment experiences

Prior treatment utilisation for alcohol and other mental health problems, and barriers to seeking treatment are examined, using a form that was developed for this study. Participants select from a list of 11 different types of providers (eg, alcoholics anonymous, family services or social service agency, inpatient ward) they may have seen related to their drinking or emotional problems, or both in the past 12 months. Participants are also asked to indicate from a list of options potential barriers for not seeking treatment for alcohol problems and mental health problems.

#### Post-IVR Survey

The Post-IVR Survey is an 11-item survey designed to assess behaviour and attitudinal change (reactivity) that may have occurred over the course of 28 days in response to IVR monitoring and was designed expressly for this study. Reactivity was not an intended goal of the study; however, it could be an important piece of measurement bias impacting outcomes. Items query about the extent to which participants may have become more aware of certain behaviours, the degree to which they may have purposefully started to make changes to their behaviour (and the specific behaviours associated with those changes), as well as feasibility and satisfaction of completing the daily surveys (length of time to complete each survey, burden of monitoring schedule, degree of difficulty completing the surveys, etc).

### IVR measurement and procedure

For 28 days following the BL, participants report their daily alcohol use, cigarette smoking, SE to quit smoking, and cravings to smoke and drink in twice daily interviews that are happen in response to two random prompts per day (eg, calls to their telephone). Prompts are programmed to coincide with the participant's sleep-wake cycle, during each of the two 4 h time intervals, one in the morning and the evening. The IVR system is configured to call (prompt) the participant's telephone and is enabled such that participants may directly access the survey after they receive the call by pressing ‘1’ on their telephone keypad. Prompting lasts for 10 s, and the participant has 2 min to respond. If the prompt is missed, the IVR system cues another prompt 5 min later. If the participant does not respond after the third prompt (15 min after the initial prompt), the trial is recorded as missed. The IVR system is set up so that no random prompts are issued within 2 h of each other; separate morning and evening interviews are programmed to facilitate different questions at each time period. IVR interviews are last 7–10 min, are date and time-stamped, and recorded immediately.

Daily factors are measured in three areas: (1) *Drinking*, including (a) frequency of beer, wine and liquor consumed in standard drink conversions and (b) quantity of the same; (2) *Smoking*, measured as number of cigarettes consumed since the prior assessment; and (3) *SE to quit smoking*, adapted from the SSE (0 ‘Strongly disagree’ to 4 ‘Strongly agree’). Factors known to correlate with drinking, smoking and SE (stressful events, mood and cravings) are also included in the daily assessments.

Several system features are enabled to promote adherence, including clear prompts, minimal skip outs, ability to return to questions, time and date stamping, and reminder phone calls from study personnel if calls are recorded as missed for one full day.^44^

To enhance IVR compliance, participants receive $15 per week for the IVR phase and they can earn additional bonus incentives of $2 per week for completing prompts for 6 of 7 days, or $5 per week for completing prompts for all 7 days of the week. Thus, participants may receive up to $108 if they complete all IVR interviews. Participants are given the option of receiving IVR payment in cash, cheque or gift card (to a local convenience or grocery store).

### Follow-up assessments

#### 1-month follow-up

Participants are contacted at the end of the daily monitoring phase for a 10 min interview of smoking status, desire to change smoking and drinking, SE to quit smoking and potential reactivity (changes in behaviour) to the IVR assessment. Participants have the option of completing the interview by phone, in-person or via the web. Participants are paid $15 for the 1-month follow-up.

#### 6-month follow-up

Participants are contacted for a final follow-up 6 months post-BL for an in-person assessment of smoking status (self-reported 7-day point prevalence abstinence) and other factors. When possible, self-reported abstinence is verified by carbon monoxide analysis of breath samples (10 ppm cut-off) for stated abstinence of 24 h–2 weeks. Detected values above the stated cut-off scores will be considered indicative of smoking.

In addition to abstinence outcomes, we also assess separate smoking behaviour change outcomes, defined as an altering of smoking behaviour with the intent or mandate to reduce or abstain, based on reports of one (or more) of the following (dichotomous yes/no): (1) reduced smoking by 50%, (2) attempt at cessation lasting more than 1 day and less than 7 days, and (3) last month continuous abstinence from smoking (yes/no). We also include duration (number of days/weeks) of cessation attempts and type of intervention/treatment utilised (nicotine gum, patch and medication). We also reassess alcohol and cigarette consumption, drug use and other tobacco product use (in the past 30 days), diagnostic status (alcohol use disorder and nicotine dependence), desire to change alcohol and smoking, SE to quit smoking, nicotine dependence and treatment utilisation for alcohol problems in the past 6 months. Participants who do not engage in cessation attempts or who increase their smoking are asked to identify and rate the degree to which certain barriers may have influenced their behaviour.

Participants who are difficult to reach may complete follow-up surveys via mail, web or over the telephone. We are not able to verify smoking status for these individuals unless they are able to come to the study site.

### Design considerations

We considered several alternative designs for this study. We had considered using a 12-month follow-up to maximise the possibility of capturing behaviour change. However, once we mapped out participant flow for this design, it did not seem feasible within the 2-year time limit of the grant mechanism. Studies show that up to 60% of smokers will make significant changes to their cigarette consumption or desire to quit in as little as 6 months.^44–46^ We have selected a sample that is high on desire to change to increase the likelihood that we will detect some change.

We considered adding a no-IVR group to control for the possibility that monitoring may cue people to reduce their drinking or smoking (ie, reactivity). However, research indicates that reactivity is highly unlikely, especially when multiple behaviours are being monitored, as is the case in this particular study.^25^ ^47^ ^48^ We will include recommended analytical ‘checks’ of reactivity^48^ and other questions on our IVR survey, aside from those about smoking and drinking to dilute the potential impact of daily recording.^49^ As one additional method to assess for reactivity, at the conclusion of the IVR phase, the participants are queried about IVR's potential role in stimulating behaviour change.

## Analyses

### Outcomes

*Primary outcome*: The main outcome analyses will be based on abstinence for at least 7 days prior to the 6-month follow-up. Self-reported abstinence reported at 6 months will be verified in person by carbon monoxide analysis of breath samples (10 ppm cut-off), if available.

*Secondary outcomes*: Secondary outcomes will be based on reports of smoking and drinking behaviour, SE to quit smoking, nicotine dependence, and motivation to change drinking and smoking collected at 1 and 6 months post-BL. We will also include separate smoking behaviour change outcomes (reduced cigarettes per day, cessation attempts and use of NRT) and barriers to change smoking as secondary outcomes.

### Sample size

Sample size projections for the daily diary analysis were based on small to medium effect sizes from prior cross-sectional and longitudinal studies between the predictors of interest (alcohol use, SE) and smoking. We are well powered to detect adequate effect sizes for all within-person/daily associations, assuming an 85% IVR compliance rate (based on daily diary studies with similar methodologies and samples^44^ ^50–56^). Power analysis of the 1 and 6-month follow-ups were based on results from naturalistic studies of smoking in ‘untreated’ samples, indicating that between 20% and 40% of smokers will reduce their daily cigarette consumption or quit completely, and another 20% will increase their motivation to change in as little as 6 months.^46^ ^57–61^ We assume a 15% attrition rate at the 6-month follow-up (N=71), based on longitudinal studies with smokers using a similar follow-up period,^31^ and we expect that approximately 60% of our study sample (n=43) will engage in some form of change by 6 months. G*Power 3.2.1 revealed that 84 participants would provide 0.80 power (α=0.05) to detect clinically significant health gains (a medium effect size^62^) for BL and daily data derived slopes on the 1 and 6-month outcomes of interest.

### Primary analyses

We will first assess patterns of missing data, attrition rates, distributional properties of dependent and other measures and correlations among all measures. Analysis of IVR data will use hierarchical linear modelling (HLM).^63–65^ HLM provides flexibility in handling missing data^65^ even when data are missing at random (MAR).^66^ ^67^ Models for aim 1 will examine the within-person daily association between drinking and smoking (drinking-smoking concordance). The within-person slope capturing drinking-smoking concordance will be saved in SPSS, and a regression model (continuous or binary logistic depending on the outcome variable) will be conducted to predict the 1 and 6-month outcome of interest from the within-person slope. Models for aim 2 will examine effects of drinking, smoking and the drinking×smoking interaction at time t (eg, morning) predicting SE at time t+1 (eg, morning predicting evening). All models will control for levels of drinking and smoking occurring in the previous interval (t−1), day of the week, and other relevant BL and demographic factors. The drinking×smoking term captures the association between smoking and SE as a function of higher or lower levels of alcohol use. Estimates of within-person slopes will be saved and imported into SPSS. Next, regression models (continuous or binary logistic) will predict the 1 and 6-month outcomes of interest from the within-person slopes (all models will control for mean levels of drinking and smoking). Mediation will be reflected by a reduction in the association between drinking-smoking concordance and smoking outcomes after including the drinking×smoking interaction on daily SE slope. To reduce the set of covariates, variables with p<0.15 will be retained in final models. To address missing data, we will control for potential variables related to missing data, as well as examine multiple imputation methods for handling missing data (expectation maximisation algorithm).^48^ ^63^^–^66

### Secondary/exploratory analyses

Effects of drinking on smoking over the course of hours (morning to evening), days or weeks (weekends vs weekdays) will be assessed. We will also examine changes in SE over the course of hours or days (creating an average change score for each person) and within-person variability in SE by calculating the SD of SE for each person.

## Ethics and dissemination

Explicit informed consent is obtained from each individual prior to participation in the study. All participants are informed that they may withdraw from the study at any time without penalty and are be reimbursed for the portion of the study that they have completed up to that point. All participant data will be kept confidential and will be identified only be a unique identifier.

Even though this is a low risk observational study, because the sample is comprised of heavy drinkers, there are several important ethical and safety issues. First, to provide additional safeguards to data confidentiality, we have obtained a CoC from the National Institutes of Health to protect participant identities and disclosure of illegal activity (drinking) for those under the age of 21. Given the high comorbidity of smoking and alcohol use with other mental health disorders, participants are given referrals to nearby treatment facilities if requested or deemed appropriate. Study staff have on-hand a list of referral sources to provide participants if they are interested.

Manuscript and conference submissions will assist with dissemination of results from this study and will provide the necessary preliminary evidence to enhance grant application success to fund a future treatment development study.

## Discussion

Major improvements in helping smokers quit will be found in the development of specialised treatments that capitalise on altering the ‘momentary’ association among risk factors that maintain smoking. Thus, findings from this proposal should provide useful pilot data for developing a low cost, minimally invasive intervention that could be used to increase smoking related SE in risky-drinking smokers, and deliver this intervention ‘in real time’ and on a platform that is free and easily accessible: one's cell phone.

## Study status

Study recruitment began in February 2014. The target sample size is N=84. As of the time of this submission (October 2014), 404 participants had been screened for the study and 63 had completed the BL assessment and been enrolled in the IVR. Recruitment is expected to be completed by February 2015.

## Supplementary Material

Reviewer comments

## Footnotes

i We had initially excluded individuals who used other tobacco products. However, on initial screening, we found that a large percentage of smokers who responded to our screening advertisement for ‘smokers who regularly drink’ did report using other tobacco products, such as little cigars/cigarillos or large cigars. As a result, we relaxed this exclusion criterion. We will examine other tobacco product use as a potential covariate in analyses.
