# Supplementary material for: Real-time patterns of smoking and alcohol use: an observational study protocol of risky-drinking smokers
Source: BMJ Open. 2015 Jan 6;5(1):e007046. doi: 10.1136/bmjopen-2014-007046 (PMC4289730; doi:10.1136/bmjopen-2014-007046)
Supplement: Reviewer comments [file bmjopen-2014-007046.reviewer_comments.pdf]

PEER REVIEW HISTORY

BMJ Open publishes all reviews undertaken for accepted manuscripts. Reviewers are asked to complete a checklist review form (**http://bmjopen.bmj.com/site/about/resources/checklist.pdf**) and are provided with free text boxes to elaborate on their assessment. These free text comments are reproduced below.

ARTICLE DETAILS

**TITLE (PROVISIONAL)**	Real-time patterns of smoking and alcohol use: An observational study protocol of risky-drinking smokers
**AUTHORS**	cohn, amy; Brandon, Thomas; Armeli, Stephen; Ehlke, Sarah; Bowers, Molly

This article was not externally reviewed at BMJ Open. Protocols that have been independently assessed prior to submission to BMJ Open are usually fast-tracked to publication on the grounds that further substantial changes will not be possible. This independent assessment will usually be external, independent review for both a substantial grant award from a non-commercial or government funder and ethics approval.
